# Intra-domain phage display (ID-PhD) of peptides and protein mini-domains censored from canonical pIII phage display

**DOI:** 10.3389/fmicb.2015.00340

**Published:** 2015-04-28

**Authors:** Katrina F. Tjhung, Frédérique Deiss, Jessica Tran, Ying Chou, Ratmir Derda

**Affiliations:** Department of Chemistry, Alberta Glycomics Centre, University of AlbertaEdmonton, AB, Canada

**Keywords:** M13 bacteriophage, phage display, protein engineering, *in vitro* selection, excised peptide

## Abstract

In this paper, we describe multivalent display of peptide and protein sequences typically censored from traditional N-terminal display on protein pIII of filamentous bacteriophage M13. Using site-directed mutagenesis of commercially available M13KE phage cloning vector, we introduced sites that permit efficient cloning using restriction enzymes between domains N1 and N2 of the pIII protein. As infectivity of phage is directly linked to the integrity of the connection between N1 and N2 domains, intra-domain phage display (ID-PhD) allows for simple quality control of the display and the natural variations in the displayed sequences. Additionally, direct linkage to phage propagation allows efficient monitoring of sequence cleavage, providing a convenient system for selection and evolution of protease-susceptible or protease-resistant sequences. As an example of the benefits of such an ID-PhD system, we displayed a negatively charged FLAG sequence, which is known to be post-translationally excised from pIII when displayed on the N-terminus, as well as positively charged sequences which suppress production of phage when displayed on the N-terminus. ID-PhD of FLAG exhibited sub-nanomolar apparent K_d_ suggesting multivalent nature of the display. A TEV-protease recognition sequence (TEVrs) co-expressed in tandem with FLAG, allowed us to demonstrate that 99.9997% of the phage displayed the FLAG-TEVrs tandem and can be recognized and cleaved by TEV-protease. The residual 0.0003% consisted of phage clones that have excised the insert from their genome. ID-PhD is also amenable to display of protein mini-domains, such as the 33-residue minimized Z-domain of protein A. We show that it is thus possible to use ID-PhD for multivalent display and selection of mini-domain proteins (Affibodies, scFv, etc.).

## Introduction

Phage display (PhD) is a powerful method for selection of peptide ligands from a diverse, random population (McCafferty et al., 1990, 1991; Scott and Smith, 1990). The diversity and uniformity of the phage-libraries are important for selection and can impact the quality of the selection process (reviewed in Derda et al., 2011). For example, it is impossible to select a sequence from a peptide library if the sequence is not present in the original library. Censorship of specific peptide sequences from phage display was recognized just a few years after PhD technology was developed. A seminal report by Dower and co-workers described the loss of propagation efficiency of phage as the number of displayed R/K residues increases (Peters et al., 1994). Total titer decreases by over an order of magnitude for each positive charge introduced in the displayed sequence. Additional censorship studies from Makowski and co-workers identified other impeding factors such as the presence of alpha-helical motifs. Peptides that display high alpha-helical propensity are delayed in periplasmic export, leading to lower titer of phage that display such sequences (Rodi et al., 2002). Hall, Noren and co-workers (Brammer et al., 2008; Nguyen et al., 2014) identified a large number of sequences with random mutations in un-translated regions (UTR) of gene *pII*; these mutations equip clones with increased growth rate. Enrichment of these fast growing “parasite” sequences is one of the mechanisms for sequence-independent censorship/loss of diversity. We have previously shown that the censorship in phage libraries can arise even due to small differences in growth rates. For two phages with a mere 20% difference in growth rates, the ratio of phages can vary by 1000-fold after a typical round of amplification (i.e., allowing phage to propagate from 10^6^ to 10^12^ PFU) (Derda et al., 2011).

There are several genetic strategies to solve the censorship problem. For example, the introduction of suppressor mutations in the SecY component of the protein export complex in *Escherichia coli* enables export of charged sequences (Peters et al., 1994). To overcome problems with censorship in the SecY pathway, Plückthun and co-workers used the conjugation of an additional signal sequence to direct protein export through the signal recognition particle (SRP) co-translation pathway rather than the post-translational SecY pathway (Steiner et al., 2006). The combination of phagemid and helper phage is the most commonly used strategy toward production of mosaic phage that carries only one copy of protein fused to pIII. Censorship in mosaic libraries is reduced but not eliminated entirely (Dev Sidhu, personal communication); to our knowledge, no side-by-side comparison of censorships in phage and phagemid systems has been reported to date. Makowski and co-workers were among the first to show that the censorship can also be avoided by using a lytic T4 system instead of non-lytic M13 phage (Krumpe et al., 2006). The most popular non-genetic solution to censorship is to compartmentalize the amplification of libraries. Early publications from the groups of Smith (Scott and Smith, 1990) and Winter (Hoogenboom and Winter, 1992) amplified phage libraries as lawns of isolated colonies on agar, although later reports questioned the efficiency of such a method (McConnell et al., 1995). Recently, we demonstrated that amplification of libraries in monodisperse emulsion can also be used to avoid the competition between fast and slow-growing phage (Derda et al., 2010b; Matochko et al., 2012b) and avoid the undesired enrichment of fast-growing “parasite” clones in phage libraries (Matochko et al., 2014).

One of the least characterized censorship mechanisms is the excision of peptides due to post-translational proteolysis. It is difficult to characterize the excision of displayed sequences from pIII because it cannot be detected on the genetic level. Phage with cleaved peptide sequences might appear to be produced at good titer and with a rate comparable to the production rate of other clones, but in fact, they do not express the desired insert. Theoretically, many types of sequences may be censored, as described in a review by Wilson and Finlay (1998), but the only well-documented case to date is the excision of FLAG sequence from 95% of phage, as detected by Enzyme-Linked Immuno-Sorbent Assay (ELISA) (Grihalde et al., 1995). Indirect evidence for post-translational cleavage has been presented by Noppe et al. (2011) who used affinity chromatography to separate the monoclonal phage populations that display a ligand for human lactoferrin (HuLF) into phage populations that have distinct affinities for the target (Noppe et al., 2011). A Heterogeneous population of phage can be separated into homogeneous HuLF-binding populations, which differ by the copy number of HuLF-peptides displayed on phage. Re-amplification of the separated homogeneous populations yields heterogeneous populations; phage that evade affinity capture on HuLF regain the ability to be captured (i.e., re-express HuLf-binding peptide) after re-amplification. We observed similar evidence for post-translational cleavage in chemical biotinylation of phage-displayed peptides. For example, in a library of Cys-containing phage, up to 30% of phage cannot be alkylated by biotin-iodoacetamide even under optimized reaction conditions (Jafari et al., 2014). The “non-reactive” clones contain the genetic sequence CX_7_C, but the peptide sequence in these phage particles is most likely cleaved during phage production.

To solve the above problems, we turned our attention to early reports of Smith (1985) and de la Cruz et al. (1988) who displayed foreign sequences between the C-terminal and N-terminal domains of pIII rather than on the N-terminus of the pIII (NT format) as it is done in modern fusion vectors. Display in between the domains was also the basis of selectively-infective phage (SIP) technology developed by Plückthun group (Krebber et al., 1997), and SAP-technology (Selection and Amplification of Phage) pioneered by Borrebaeck group (Duenas and Borrebaeck, 1994; Nilsson et al., 2002). All reports rest on the fundamental observation that M13 phage can tolerate the integration of large foreign sequences in between the domains of the pIII protein. For example, introduction of the β-lactamase protein (approximately 30 kDa) can yield infective phage, albeit the efficiency of the production of phage dropped by a factor of ten thousand from 10^11^ PFU/mL to 10^7^ PFU/mL (Krebber et al., 1997). Other insertions, such as RNAseT1 can also be tolerated (Sieber et al., 1998b). Inserted sequences are accessible to protein binding and can generate immune responses when phage are injected into mice (de la Cruz et al., 1988).

In this report we assess whether the insertion of short to medium-size peptide sequences between the domains of pIII in mature phage can help mitigate the problems listed above: (i) permit display of charged peptides and sequences with alpha-helical propensity; (ii) prevent loss of sequences due to post-translational proteolysis; (iii) allow direct quantification of sequences that retain the displayed sequence during production. The latter requirement is possible because the loss of displayed sequence is manifested as a loss of infectivity (Sieber et al., 1998a). Investigation of insertion of fragments of larger size is another important question; however, we focus our current report on peptide sequences of 4–40 amino acids in length. To develop a platform complementary to the fd-tet-based platform used by the Plückthun group in the 1990's (Krebber et al., 1997), we built the intra-domain phage-display (ID-PhD) on the well-established and commercially available M13KE platform (Noren and Noren, 2001), which is one of the most utilized phage-display platforms: it was used to identify over 2000 ligands to-date according to the MimoDB 2.0 and 3.0 databases (http://i.uestc.edu.cn/mimodb/index.html) (Huang et al., 2012). As described in the results, the ID-PhD phage can be propagated, enumerated and tested in conditions analogous to those developed for M13KE phage. Here, we describe successful insertion and expression of seven functional peptide sequences that are typically censored from traditional phage display: (i) the highly-charged FLAG peptide sequence DYKDDDDK; (ii) a miniaturized triple-helical Z-domain of the IgG-binding protein A (mZ) composed of 33 residues (Braisted and Wells, 1996), and (iii–vi) four positively charged arginine-containing sequences, termed “RAE”-sequences, previously shown to impede phage amplification (Peters et al., 1994). Additionally, we cloned a library of random tetrapeptides into the ID-vector for future studies of censorship in ID vs. NT format.

## Materials and methods

### Bacterial strains, culture conditions, and plasmids

ID1-PhD and ID2-PhD vectors were engineered from a phage clone displaying the sequence H_2_N-SVEKNDQKTYHA originating from a Ph.D.-12 phage display library based on the filamentous bacteriophage vector M13KE (New England BioLabs, Ipswtich, MA, USA). The propagation host *E. coli* K12 ER2738 was maintained on LB agar plates with 20 μg/mL tetracycline. The double-stranded replicative form DNA phage plasmids were collected from log-phase cultures of *E. coli* grown in the presence of phage for up to 6 h in LB medium at 37°C with shaking. Cultures were processed using the GeneJET Plasmid Miniprep kit (Thermo Fisher Scientific, Waltham, MA, USA).

### Engineering and construction of ID1-PhD and ID2-PhD

**Figure 1:**
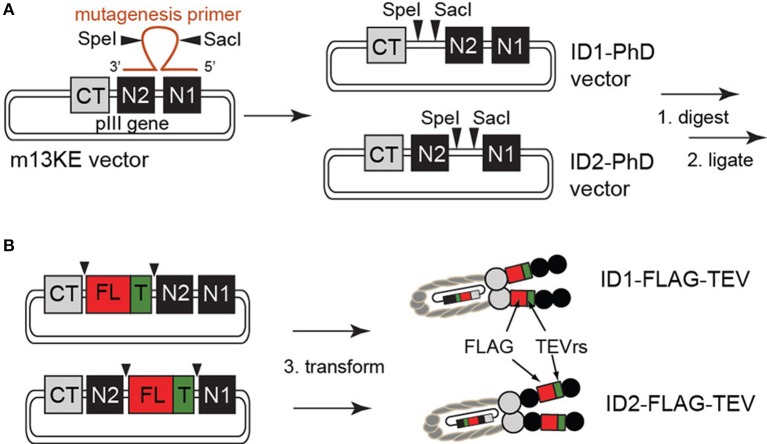
**(A)** Construction of ID1- and ID2-cloning vectors by site-directed mutagenesis. Due to the high degree of homology of the glycine-rich linker between CT–N2 and N2–N1 domains, phage clones containing SacI and SpeI sites at both locations arise from a single round of mutagenesis. **(B)** Insertion of the FLAG peptide and TEV-protease recognition site was achieved by restriction enzyme digest and subsequent ligation of a synthetic TEVrs-FLAG DNA fragment. All constructs were validated by sequencing (see Supplementary Materials).

Exogenous restriction enzyme sites for SacI and SpeI were inserted into the M13KE plasmid by site-directed mutagenesis, according to manufacturer guidelines (QuikChange II kit, Agilent Technologies, Santa Clara, CA, USA). Briefly, mutagenesis primers containing both restriction enzyme sites separated by six arbitrary nucleotides and two flanking complementary regions of 13 bases in length were used to insert SacI and SpeI sites into the M13KE replicative form (RF) double-stranded DNA template by PCR (see Supplementary Materials for primer sequences). Following degradation of the template with DpnI, mutagenized plasmids were transfected into XL1-Blue supercompetent cells and screened on LB-agar plates supplemented with isopropyl-1-thio-β-D-galactopyranoside (IPTG) and 5-bromo-4-chloro-3-indolyl-β-D-galactopyranoside (X-gal). Blue colonies were selected for sequencing. Note: We used commercial Quickchange II kit with XL1-Blue Supercompetent cells (pilus-negative); although these cells cannot propagate the M13KE phage continuously, they contain a lacZΔ M15 allele that allows identification of the transformants by complementing the β-galactosidase gene with the LacZα supplied by M13KE vector. We did not supplement our agar plates with kanamycin or ampicillin because M13KE does not confer antibiotic resistance; therefore, in a typical experiment the vast majority of the colonies were non-transformed (white) and rare transformed variants were identified as blue-specks in a “carpet of bacteria.” If necessary, the selected colonies were resuspended in LB and immediately re-streaked on non-selective agar plates to dilute the colonies and isolate a pure monoclonal LacZ(+) population for sequencing. Alternatively to streaking on non-selective plates, we mixed the transformed XL1-Blue cells with F(+) *E. coli* K12 ER2738 an hour after heat shock transformation and plated in an agar overlay to select the M13KE-transformants as blue plaques. Sequencing data showed two independent products that contained SacI and SpeI recognition sequences (see Figure 1 and sequencing data in Supplementary Materials). Successful insertion was also confirmed by SacI/SpeI digestion of the dsDNA.

### Cloning of FLAG, mZ, RAE-sequences, and TEV-recognition sequences (TEVrs) into ID-PhD vectors

Synthetic single-stranded DNA (ssDNA) containing FLAG-TEVrs, “RAE” sequences, and mZ-TEVrs inserts were purchased from Integrated DNA Technologies (Coralville, IA, USA); sequences were designed with flanking SacI and SpeI recognition sequences complementary to the mutagenized phage vector to enable restriction enzyme cloning (see Supplementary Materials for sequences). Inserts were converted from ssDNA to dsDNA by PCR amplification of the synthetic DNA forward strand. After ethanol precipitation, restriction enzyme digestion of the mutagenized phage plasmid (1 μg per reaction) and the insert duplex (0.2 μg per reaction) was performed using 1 U each of SacI and SpeI enzymes in the provided Green Buffer at 37°C for 30 min (FastDigest Restriction Enzymes, Thermo Fisher Scientific, Waltham, MA, USA). Digested vector was purified by agarose gel purification followed by gel extraction by GeneJET Gel Extraction Kit (Thermo Fisher Scientific, Waltham, MA, USA), or DNE elution from agarose using Elutrap® Electroelution System (GE Healthcare/Whatman) run at 100 V for 3–9 h, followed by ethanol precipitation. Digested insert was purified using E-Gel® SizeSelect™ Agarose Gel, 2% (Life Technologies, Carlsbad, CA, USA) according to the instructions of the manufacturer. Test ligation reactions with plasmid-to-insert ratios of 1:1, 1:3, or 1:10 and T4 DNA Ligase were incubated overnight at 16°C (New England BioLabs, Ipswitch, MA, USA). Approximately 5 ng of ligated product was transformed into 50 μL of chemically competent *E. coli* K12 (produced in-house by RbCl_2_ method). This low efficiency method is suitable for production of individual clones with ID1/ID2-inserts. A 10 μL sample of the transformed culture was immediately spread on LB-agar plates either undiluted or at a 100-fold dilution (*see note about spreading in* Section Engineering and Construction of ID1-PhD and ID2-PhD); alternatively, it was plated in an agar overlay supplemented with F(+) *E. coli* K12 ER2738 to select the transformants as blue plaques. For more efficient transformation and cloning of libraries of peptides in ID-format, we electroporated the ligated constructs using the Gene Pulser Xcell™ Electroporation System and 2 mm GenePulser/MicroPulser Electroporation Cuvettes (all from BioRad) with the settings 2500 V/400 Ohm/46 μF using commercially available F(+) TG1 electrocompetent cells and recovery media (Lucigen, Middleton, WI, USA). Where necessary, we quantified the number of transformants by mixing the cells exactly 30 min after transformation (i.e., before generation of the first progeny) with F(+) *E. coli* K12 ER2738 and plating the mix immediately in the agar overlay on X-gal/IPTG plates. We found the best plasmid-to-insert ratio to be 1:10. Individual colonies or plaques (*n* = 8) were picked and amplified in LB medium supplemented with 1:100 dilution of log-phase culture of *E. coli* K12 ER2738 for plasmid DNA isolation. Sanger DNA sequencing confirmed the incorporation of the insert (Supplementary Materials). While in this report we used restriction ligation, sequences can be cloned into the ID-vector using other techniques, such as Kunkel mutagenesis (Scholle et al., 2005).

As a control, we cloned a FLAG-TEVrs sequence (see Supplementary Materials) into the N-terminus of the pIII protein in the M13KE vector (NT-FLAG) using KpnI and EagI restriction sites and a standard protocol adapted from Ph.D.™-12 Phage Display Peptide Library Kit (New England Biolabs, Ipswitch, MA, USA) and using electroporation and TG1 electrocompetent cells as described above. All constructs were validated by Sanger sequencing (Supplementary Materials).

### Functional validation of ID1-FLAG and ID1-mZ

**Figure 2:**
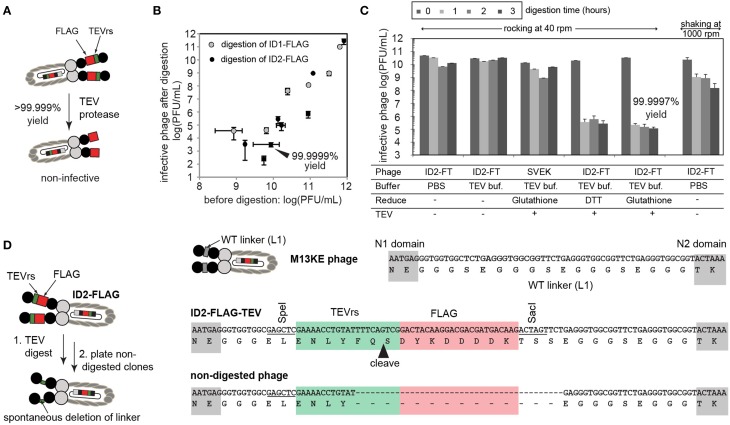
**(A)** The presence of inserted TEV sequence can be validated by enzymatic digestion of the phage and by measuring the number of infective particles before and after digestion by TEV-protease. **(B)** Under optimized conditions (dilute concentration of phage), digestion efficiency can reach an apparent 99.9999%, indicating that the majority of phage display the insert. Each point describes a separate digestion experiment. Error bars describe standard deviation of titering. **(C)** Optimization of buffer, reducing agents, and mixing conditions used for digestion. Interestingly, simple vigorous shaking at 1000 rpm leads to drastic loss of infective particles. With gentle rocking, we observed a minor decrease in the number of infective particles due to mild toxicity of the reducing agent. Under conditions that minimize non-specific toxicity, phage is digested by TEV in <1 h. Prolonged incubation does not improve digestion beyond 99.9997% yield. Error bars represent standard deviation between three independent experiments. **(D)** Clonal culture and sequencing of the residual 0.0003% phage revealed that non-digested phage lost the insert due to genetic deletion. Note that these phage are not the result of contamination (e.g., they are clearly different from “environmental” M13 phage) as they retain fragments of TEVrs and SacI/SpeI recognition sequences.

To confirm complete expression of FLAG-TEVrs and mZ-TEVrs amino acid sequences, we performed enzyme-digestion and ELISA-based assays. To validate the expression of the TEVrs sequence ENLYFQS, ID1-FLAG, ID2-FLAG, and ID1-mZ phage were treated with TEV protease, according to manufacturer instructions (ProTEV Plus, Promega, Madison, WI, USA). Briefly, approximately 10^11^ PFU/mL of phage was added to 1x of the provided reaction buffer (100 μL) supplemented with 1 mM dithiothreitol (DTT). The mixture was incubated at room temperature (r.t.) with shaking or gentle rocking for 1–3 h (Figure 2). Input and output phage were titered using a standard agar overlay method with *E. coli* K12 ER2738.

**Figure 3:**
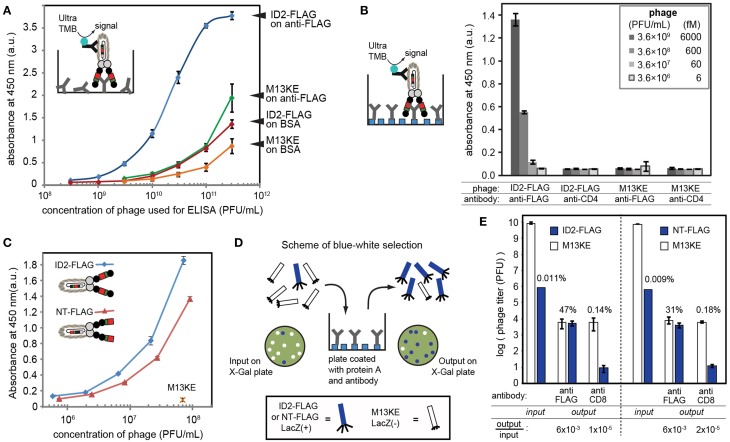
**(A)** The presence of inserted FLAG sequence can be validated by ELISA on wells coated by anti-FLAG antibody. Phage was detected using HRP-conjugated anti-M13 antibody and Ultra-TMB substrate for horseradish peroxidase (HRP). In control experiments, we coated wells with BSA or used M13KE phage without a FLAG insert. **(B)** ELISA signal in optimal conditions with oriented immobilization of anti-FLAG IgG on protein A-coated wells yielded signal detectible at 600 femtomolar concentration of phage (3.6 × 10^8^ PFU/mL). In this experiment, we used wells coated by non-specific IgG (anti-CD4) as negative control. **(C)** Comparison of phage displaying FLAG at the N-terminus (NT) and in intra-domain (ID) fashion using setup analogous to **(B)**. ID-display provides statistically significantly higher signal at equivalent titers. **(D)** Scheme of selection of ID-FLAG or NT-FLAG from a 1:10,000 mixture with insert-free phage. Enrichment is monitored as increase of blue (LacZ+) plaques on agar overlay containing X-gal. **(E)** Results of selection of ID-FLAG or NT-FLAG on wells presenting anti-FLAG or anti-CD8 (negative control). ID-display provides minor yet detectable advantage. Input to output ratios for insert-containing clones are displayed below the plot. In all experiments, data represents average from three to four independent wells; error bar is one standard deviation.

**Figure 4:**
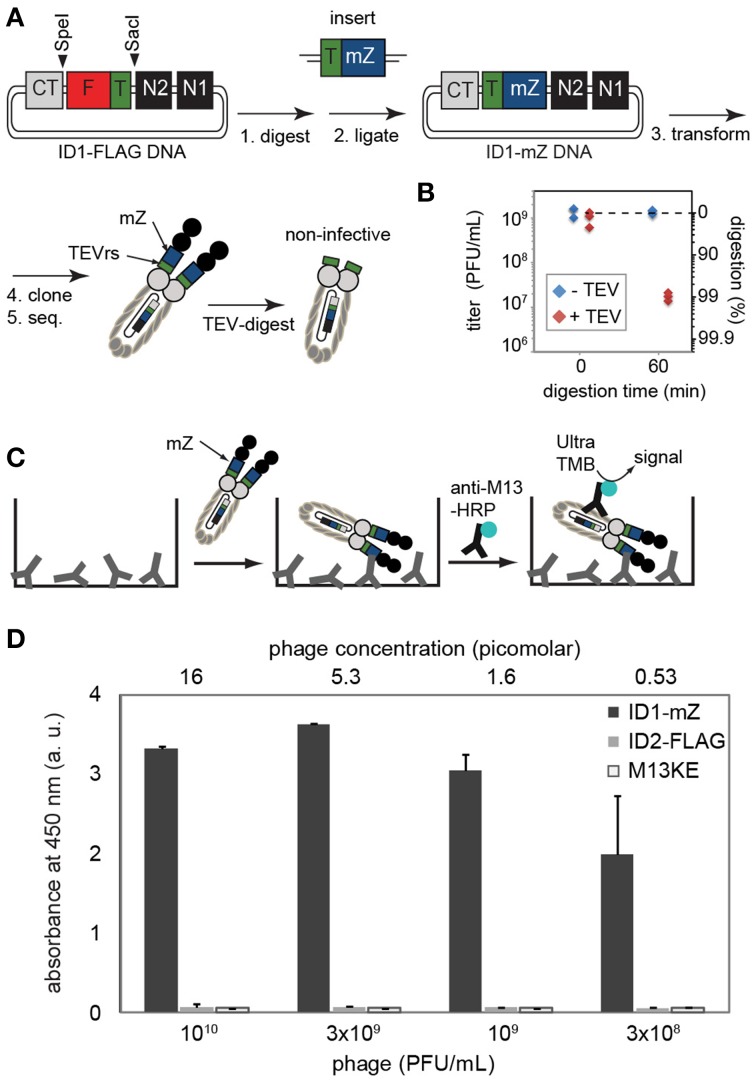
**(A)** Steps toward displaying mZ protein and TEV recognition sequence. The complete sequence of the cloned construct is provided in Supplementary Materials. **(B)** Digestion by TEV-protease validates that at least 99% of phage contained the insert and lost infectivity in the presence of this protease. Phage do not lose infectivity in a solution that has all components (reducing agents, buffers, salts) but no TEV-protease. **(C,D)** The presence of inserted FLAG sequence can be validated by ELISA on wells coated by IgG antibody that binds to the mZ domain. Phage were detected using HRP-conjugated anti-M13 antibody and Ultra-TMB substrate for HRP. ID2-PhD phage that displayed FLAG sequence or original M13KE phage that displayed no sequence exhibited no binding to IgG-coated wells. Data represents average from three independent wells; error bar is one standard deviation.

We used immunoassays to validate complete expression of FLAG peptide sequence DYKDDDDK and the miniaturized Z-domain of protein A by ID2-FLAG, NT-FLAG and ID1-mZ phage, respectively. In the case of ID2-FLAG and NT-FLAG (Figure 3A), we coated the wells of a 96-well flat-bottomed plate (Corning Life Sciences, Tewksbury, MA, USA) with 100 μL of a 1 μg/mL solution of anti-FLAG mouse IgG (GenScript Piscataway, NJ, USA) in PBS overnight at 4°C. In the case of ID1-mZ (Figure 4), the coating antibody was mouse anti-human CD4 IgG (BD Biosciences, San Jose, CA, USA) at a concentration of 4 μg/mL. Following a 1 h blocking step with 3% w/v BSA in PBS, solutions of ID1-mZ phage (3 × 10^8^ to 1 × 10^10^ PFU/mL), ID2-FLAG-TEV phage or TEV protease-digested ID2-FLAG phage (3 × 10^8^ to 3 × 10^11^ PFU/mL) in binding buffer (0.1% w/v Tween and 0.1% w/v BSA in PBS) were added and incubated for 1 h at r.t. Following a wash step (washing buffer: 0.1% w/v Tween in PBS, 200 μL × 4 washes), 100 μL of 1:5000 dilution of HRP-conjugated anti-M13 phage antibody (GE Healthcare, Pittsburg, PA, USA) was added and incubated for 1 h at r.t. After another wash step, 100 μL of 1-Step Ultra TMB ELISA Substrate was added for 30 min, followed by a quenching step with 100 μL of 2 M sulfuric acid. Absorbance at 450 nm was then recorded.

**Figure 5:**
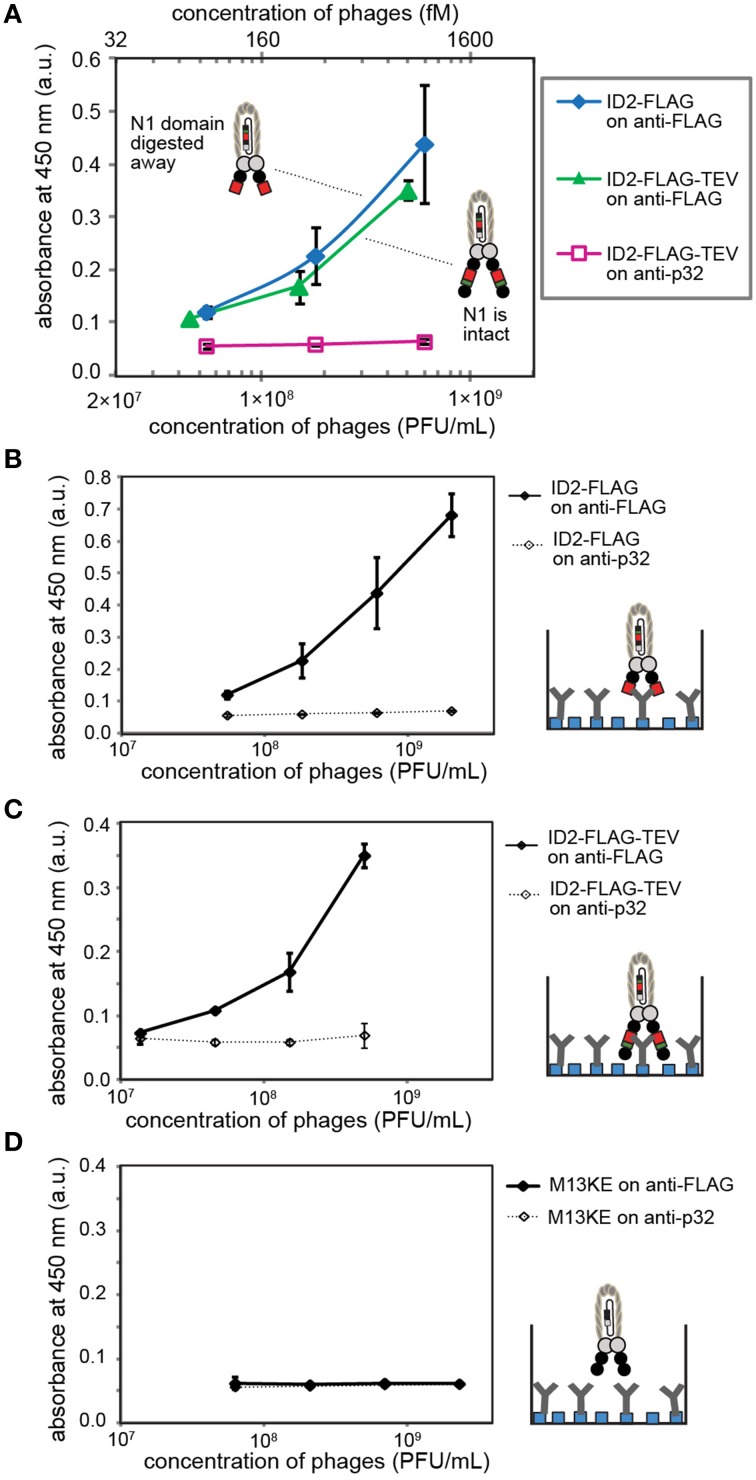
**(A)** ELISA detects inserted FLAG sequences before and after digestion of ID2-FLAG-TEVrs phage by TEV protease. When tested side-by-side, digested and non-digested phage exhibit similar binding response. This observation suggests that the N2 domain does not interfere with binding of anti-FLAG antibody. **(B–D)** Control experiments in which we compared **(B)** binding of digested phage to matched (anti-FLAG) and mismatched (anti-p32) antibody; **(C)** binding of undigested phage to matched/mismatched antibody; **(D)** binding of insert-free phage to matched/mismatched antibody. Antibodies were immobilized on protein-A coated wells. Data represents average from three independent wells; error bar is one standard deviation.

Oriented display of anti-FLAG antibody (Figures 3B, 5) was achieved by first coating wells with 100 μL of 0.32 μg/mL (10 nM in PBS) of a fusion protein that contains IgG-binding mZ domain. The wells were then blocked for 1 h with 200 μL of 2% w/v BSA in PBS and washed using a plate washer (BioTek Plate Washer 405 TS) which repeated the following program 10 times: (i) add 200 μL/well of washing buffer; (ii) shake for 10 s; (iii) soak for 15 s; (iv) aspirate each well. After adding 50 μL of antibodies in binding buffer at a concentration of 4 μg/mL for anti-FLAG and anti-CD4 antibodies, and 2 μg/mL for anti-p32 (EMD Millipore, Temecula, CA, USA), the plate was incubated for 30 min at r.t. After another washing step with the plate washer, 50 μL of ID2-FLAG-TEV phage, ID2-FLAG phage and M13KE phage in binding buffer were added and incubated for 30 min. The unbound phages were washed away using the plate-washer procedure and each well was then incubated for 30 min at r.t. with 100 μL of HRP-conjugated anti-M13 antibody supplemented with protein A at a concentration of 100 μg/mL (Figure 3B) or 30 μg/mL (Figure 5). The addition of protein A to the solution of anti-M13 antibody was necessary to prevent non-specific binding of anti-M13 antibody to the surface of the plate. Following a final washing step with the plate washer, 50 μL of 1-Step Ultra TMB ELISA Substrate was added and quenched after 15 min with 50 μL of 2 M sulfuric acid. In all ELISA experiments, three technical replicates were used unless specified otherwise.

### Panning of ID2-FLAG and NT-FLAG on anti-FLAG

We coated a 96-well high binding Costar® Assay Plate overnight at 4°C with 100 μL of protein A (Sino Biological Inc, Beijing, China) at 10 μg/ml in PBS. To test binding of ID2-FLAG and NT-FLAG on anti-FLAG, wells were coated with anti-FLAG at 1.5 × 10 ^−3^ mg/mL, while wells for control experiments were coated with 1.5 × 10^−3^ mg/mL anti-human CD8 (BD Pharmingen™, San Jose, CA, USA) for 1 h at r.t. Wells were washed 10 times using a plate washer, programmed as follows: (i) dispense 300 μL/well of washing buffer (50 mM MOPS, 150 mM NaCl, 2 mM CaCl_2,_0.1% w/v Tween, pH 7.4); (ii) shake for 5 s; (iii) soak for 30 s; (iv) aspirate each well. Next, two libraries were prepared in binding buffer (50 mM MOPS, 150 mM NaCl, 2 mM CaCl_2_) for panning: (1) ID2-FLAG mixed with LacZα(-) M13KE (“WT”) (Derda et al., 2010a) at a ratio of ~1:10,000 (9 × 10^6^: 9 × 10^10^ PFU/ml); and (2) NT-FLAG mixed with “WT” at a ratio of ~1:10,000 (6 × 10^6^: 8 × 10^10^ PFU/mL). Libraries (100 μL) were distributed each into a separate well, resulting in four replicates of each library in wells coated with anti-FLAG, and three replicates of each library in wells coated with anti-human CD8 (control). Following 1 h incubation at r.t. and another washing step (10 cycles as described above), each well was eluted with 200 μL of elution buffer (0.2 M glycine-HCl, 0.1% w/v BSA, pH 2.2) for 9 min, neutralized with 25 μL neutralization buffer (1 M Tris-HCl, pH 9.1) and titered using an agar overlay on LB X-Gal/IPTG plates.

## Results

### ID1-PhD and ID2-PhD cloning vectors

Figure 1 summarizes the structure of the pIII protein of M13 phage as C-terminal (CT) and two N-terminal (N1 and N2) domains separated by two linkers. The vectors constructed from this phage were termed ID1 if they contained the cloning sites in the first intra-domain region L1 (between CT and N2) or ID2 if the sites were in the second intra-domain region L2 (between N2 and N1). We used site-directed mutagenesis to introduce exogenous SacI and SpeI restriction enzyme sites between CT and N1 domains of a phage clone displaying H_2_N-SVEKNDQKTYHA on the terminus of the N2 domain (hereafter referred to as M13KE phage) (Ng et al., 2012). This clone originated from the commercially available Ph.D.-12 phage display library (New England BioLabs, Ipswitch, MA, USA). While our mutagenesis primers were designed to anneal to the L1 linker, due to high redundancy between L1 and L2, we also isolated mutants that incorporated the SacI-SpeI insert in L2. These two mutants are the basis of ID1-PhD and ID2-PhD cloning vectors. Insertion of the desired displaying sequences into these vectors is accomplished by straightforward one-pot restriction digestion of vector and synthetic insert by SacI/SpeI mixture and “sticky-end ligation” of the SacI and SpeI complementary ends.

### Construction and validation of ID2-TEV-FLAG

As an initial test, we cloned an insert that contained both the FLAG peptide sequence DYKDDDDK and TEV-protease recognition (TEVrs) sequence ENLYFQS into cloning vectors ID1-PhD and ID2-PhD. Presence of 15 foreign amino acids with eight charged residues did not influence the growth of phage or the titer of the phage. The original M13KE, the phage expressed from cloning vectors ID1-PhD or ID2-PhD and the resulting ID1-FLAG or ID2-FLAG phage were able to reach a similar titer (10^12^ PFU/mL after overnight culture in *E. coli* K12). We note that ID(1/2)-FLAG phages are formally a double-display platform because they contain a 15-mer intra-domain (FLAG-TEVrs) and 12-mer N-terminal display (SVEKNDQKTYHA) simultaneously.

### Quantification of displayed sequence expression using TEV-protease

We used enzymatic digestion by TEV-protease for detection of the insert in ID-PhD vectors. Conveniently, enzymatic digestion of TEVrs removes the N-terminal domain of phage and renders the phage non-infective (Figure 2A). Measuring the infectivity of phage before and after treatment of phage with TEV-protease is a rapid way to measure the digestion efficiency. For reasons we could not understand, digestion of phage was only 90% complete at high concentration of phage (>10^12^ PFU/mL), possibly due to inhibition of TEV-protease by the by-products of phage preparation. At lower concentrations of phage, digestion efficiency increased exponentially, reaching over 99.9999% yield at 10^10^ PFU/mL. For example, after a brief treatment of 10^10^ PFU/mL of ID2-FLAG phage with TEV protease, only ~10^3^ PFU/mL, or one in 10 million phage particles remained undigested (Figure 2B). In the same conditions, ID1-FLAG phage exhibited ~10 fold lower digestion efficiency (Figure 2B). It is important to note that even the mixing technique could result in significant non-specific decrease in the infectivity: for example, prolonged mixing of phage at 1000 rpm caused considerable loss in infectivity, while gentle rocking exhibited none (Figure 2C). To validate that this loss of infectivity is not caused by non-specific toxicity of the TEV-digestions conditions, we tested and optimized every factor in this condition to improve mixing, pH, and reducing agent. Under optimized conditions, we reproducibly observed a digestion yield of ID2-FLAG phage to be 99.9997% after 1 h, while a decrease in infectivity for all controls (TEV-free, or insert-free) was minimal (Figure 2C).

ID-PhD of protease recognition sequences provide the unique ability to measure the kinetics of enzymatic digestion of proteins present in solution at pico-, femto-, and theoretically even single-molecule concentrations. As ID-display of TEVrs links digestion to infectivity directly, it can complement existing methods that require affinity-capture of digested phage (McCarter et al., 2004; Scholle et al., 2006). We also investigated the origin of the one in 1,000,000 phage particles that appeared to be resistant to digestion. Specifically, during digestion of ID2-FLAG phage, concentration of TEVrs peptides decreased from ~60 picomolar to ~60 attomolar (10,000 non-digested particles) after 1 h. Prolonged incubation (Figure 2C) or addition of fresh TEV (not shown) could not digest the remaining 10,000 molecules. Upon inspection, we found that particles resistant to digestion were phage clones that underwent spontaneous genetic deletion of FLAG-TEVrs insert during culture. Figure 2D describes one sequence in which the rudiments of the TEVrs sequence can be recognized. Spontaneous deletions are known to occur in vectors based on filamentous phage.

The display of the tandem FLAG-TEVrs sequence system allowed us not only to confirm the presence of FLAG motif but also to measure that the rate of genetic deletions of the insert was one in 1,000,000. Out-of frame deletions can happen as well; however, these mutations are disruptive to the full translation of the pIII protein and result in the failure to terminate assembly and release the phage into the medium (Rakonjac and Model, 1998). Measurement of residual infectivity in the ID-PhD system, thus, can be used to trace the stability of the insert and its propensity toward specific deletion.

### Specificity of ID2-FLAG

We tested for the presence of the FLAG sequence using ELISA, in which we tested the binding of ID2-FLAG phage and control M13KE phage to plates coated with anti-FLAG antibody or bovine-serum albumin (BSA) (Figure 3A). ID2-FLAG exhibited significantly stronger dose-dependent binding to anti-FLAG than any of the control combinations. While ELISA provided only an indirect estimate of binding affinity, the mid-point (EC_50_) of binding of ID2-FLAG to surface-immobilized anti-FLAG can be estimated to be in the (1–3) × 10^10^ PFU/mL range or 10–60 picomolar. The reported affinity between monovalent FLAG peptide and anti-FLAG antibody immobilized on the surface is 300–400 nM as measured by surface plasmon resonance (Einhauer and Jungbauer, 2001). The apparent increase in affinity is a manifestation of the multivalent interaction between polyvalent display of FLAG and divalent anti-FLAG antibody on the surface. We note that direct comparison of affinity is impossible because techniques used to measure monovalent and multivalent binding are drastically different (SPR vs. ELISA). Nevertheless, we believe that it is the multivalent presentation of the relatively weak FLAG epitope on phage under optimal conditions that allowed us to detect specific binding of phage to oriented display of anti-FLAG antibody at concentration as low as 10^8^ PFU/mL or ~100 femtomolar (Figure 3B). None of the mis-matched controls (non-FLAG phage, non-FLAG antibody) exhibited any detectable binding at this concentration.

By design, the efficiency of display of FLAG sequence is higher in ID vs. NT-display and this factor could make ID-display more efficient in selection procedures. In the ID-PhD vector, >99.99% of **infective** particles contain FLAG because the presence of FLAG peptide is linked to infectivity. The only mechanism for removal of the ID-displayed FLAG from an infective particle is an in-frame deletion of its coding sequence; however, such excision is rare (<0.01%) based on TEV-digestion studies (Figures 2C,D). In contrast, in NT display the FLAG sequence is known to be trimmed from 90 to 95% without compromising infectivity of the particles (Grihalde et al., 1995). We tested the extent of the FLAG tag removal by direct comparison of ID-FLAG and NT-FLAG binding to immobilized FLAG-specific antibody using ELISA (Figure 3C). When FLAG peptide is presented at the N-terminus, the efficiency of such display is lower in ELISA. We also compared the efficiency of both displays in a model selection of FLAG-containing phage from a mixture with FLAG-free phage in a 1:10,000 ratio (Figures 3D,E). Conveniently, FLAG-free phage has a defective LacZα sequence and produces colorless plaques. Efficiency of selection can be assessed directly by counting blue and white plaques in an agar overlay containing X-Gal/IPTG (Derda et al., 2010a). ID-display provided minor yet detectable advantage when compared to NT-display.

### Characterization of ID1-mZ

To show that the ID-PhD system can be used to display larger poly-peptide sequences and therapeutically relevant mini-domain proteins, we expressed a minimized Z domain (mZ) of protein A. The three-helix bundle mZ domain is used as a scaffold for the popular Affibody™ technology (Nord et al., 1997). Introduction of tandem mZ and TEVrs sequences, 40 amino acids in total, into the ID1-PhD vector (Figure 4A) had a minor effect on the phage titers. Overnight culture of ID1-mZ yielded 10^11^ PFU/mL; ~10x lower than ID2-PhD phage. TEV-protease digestion confirmed presence of mZ-TEVrs insert in ~99% of phage (Figure 4B). A residual fraction of non-digested phage in a clonal population of ID1-mZ (1%) was significantly higher than non-digested phage in ID1-FLAG population (~0.001%, Figure 2B). This observation suggested that the frequency of spontaneous deletions of a 40 amino acid sequence (1 in 100) is more frequent than deletion of a 14 amino acid sequence (1 in 10^6^, Figure 2). This spontaneous deletion is likely to be the limiting factor of ID-PhD technology for display of large proteins. TEV-protease digestion of TEVrs in tandem with a peptide of interest can be used to assess display efficiency and retention of any peptide of interest in ID-format. To date, the only way to measure such efficiency was Western Blot of pIII protein with an antibody reactive to the specific sequence of interest. TEV digestion can be performed on any sequence and is conceptually simple because it requires only measurement of titer before and after addition of TEV protease.

Phage that displayed mZ protein bound specifically to the plates coated with IgG antibodies but failed to bind to the surfaces coated by control proteins (BSA) (Figure 4D). Similarly to ID2-FLAG, ID1-mZ exhibited exceptionally strong concentration-dependent binding to its receptor. We estimated the mid-point of binding (EC_50_) of ID1-mZ to surface-immobilized IgG to be in the 3 × 10^8^ PFU/mL or 600 femtomolar range of concentration of phage. The reported affinity between monovalent mZ domain and IgG antibody is 43 nM as measured by SPR (Braisted and Wells, 1996). Just as above, we caution that comparison based on EC_50_ and K_*d*_ measured by two independent methods might not be entirely accurate; nevertheless a large, apparent 50,000-fold difference between these values can be considered as strong indication for the multivalency of the ID1-mZ display.

### Prospect of ID-PhD platform for SIP technology

We tested whether deletion of the N1 domain by proteolysis of ID2-FLAG phage yields phage particles in which FLAG sequence can be recognized by the antibody. This recognition can be subsequently used in selectively-infective phage (SIP) technology for screening of peptide sequences that can restore infectivity of phage by recruiting a fusion of N1 and the protein of interest (Krebber et al., 1997). Side by side testing of digested and non-digested phage yielded indistinguishable concentration-dependent binding of phage to wells coated with anti-FLAG antibody (Figure 5A). Every control tested in this system (e.g., mismatched antibody, control phage) yielded no significant signal above background, indicating the specificity of the response (Figures 5B–D). In this experiment, TEV digestion conveniently converts ID-display to NT-like-display by removing the N1 domain. The observation that the epitope exhibits a similar binding affinity in digested and undigested phage suggests that intra-domain format does not interfere with the recognition of FLAG epitope by anti-FLAG antibody.

### Expression of positively charged sequences and library of peptides in ID format

**Figure 6:**
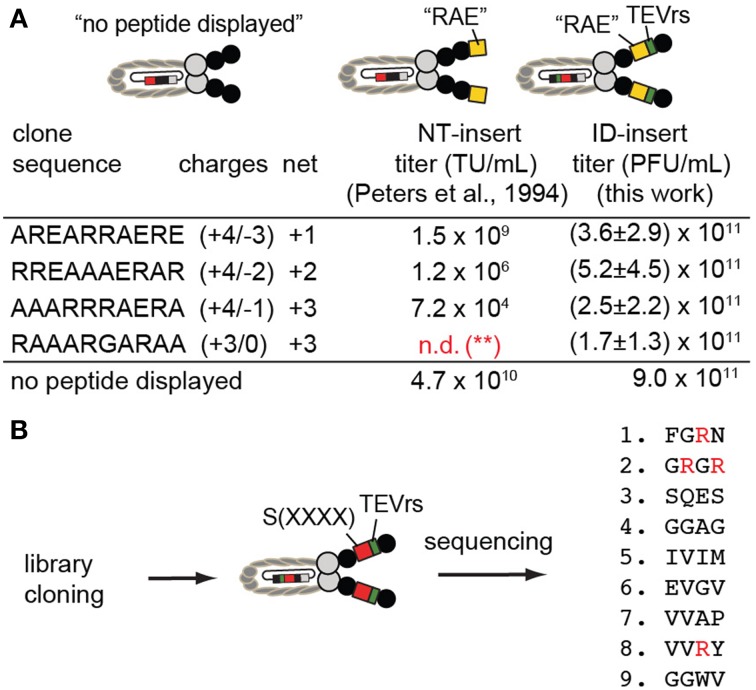
**(A)** ID-PhD enhances the expression of positively charged peptide sequences. The data in “NT-insert”-column has been acquired by Dower and co-workers (Peters et al., 1994); it shows that Arg-rich “RAE”-sequences, when expressed at the N-terminus (NT) of pIII, impede phage titer by 2–6 orders of magnitude (TU = transducing units). We demonstrated that phage clones that display these sequences in the ID-format do not exhibit diminished titers; they are similar to the titer exhibited by phage without an insert (control phage labeled as “no peptide displayed”). Note that in experiments by Peters et al. (1994), control phage is FdTet phage displaying no N-terminal sequence. In our experiments, control phage is M13KE phage without ID-insert. The data is average and standard deviation from titer in three independent experiments. (^**^): n.d., not determined; **(B)** We expressed the library of random tetrapeptides in ID-PhD format and, in preliminary low-throughput sequencing, observed a significant fraction of peptides with Arg. Abundance of Arg can be further validated in this library using next-generation sequencing.

Positively charged peptides such as AREARRAERE, RREAAAERAR, AAARRRAERA, when displayed at the N-terminus (NT) of phage, were reported by Dower and co-workers (Peters et al., 1994) to suppress formation of phage by as much as 6 orders of magnitude. Data previously collected by Peters et al. (1994), is described in columns labeled “NT-insert” (Figure 6A). We cloned the same sequences into the ID2-PhD vector (see Supplementary material for cloning and validation by sequencing); we then determined the titer after amplification of phage bearing these sequences. We observed that the production of phage with insert that contained Arg-rich sequences remained at levels compatible to the titer of the clone without the ID2-insert (Figure 6A). Specifically, the determination of the titers was conducted by mixing 3000–10,000 PFU of phage with a 1:100 dilution of log-phase culture of *E. coli* in 1 mL of LB. After overnight culture, we removed the bacteria by centrifugation and measured the number of newly produced phage in the supernatant using plaque-forming assay. For phage that displayed three Arg-residues, the titer in ID-display was several orders of magnitude higher than display at the N-terminus suggesting that presence of Arg-rich peptides is tolerated in ID-display.

We then cloned a library of sequences in ID-PhD format and analyzed a small number (9) of clones by Sanger sequencing (Figure 6B). We focused our attention on Arg residues because the abundance of Arg in NT-libraries is significantly lower than expected from the codon frequencies for this amino acid (3 codons out of 32 NNK-codons encode Arg) (Rodi et al., 2002; Matochko et al., 2012a). In X4-ID-PhD library, we observed four Arg residues in nine tetra-peptides (4 out of 36 amino acids) (Figure 6B). These preliminary observations suggest that censorship of Arg peptides might be lower in the ID-format. In the future, it will be interesting to characterize the X4-ID-library or other libraries by Illumia sequencing to confirm this observation.

## Discussion

We demonstrated that the ID-PhD system can be used to display short charged peptides and protein mini-domains of up to 40 residues. Quality control of display and its long-term stability upon serial re-growth is important in phage display, and the ID-PhD system offers robust built-in quality control based on linkage of display and phage infectivity. For example, TEV protease-digested and residual non-digested phage can be used to measure the exact fraction of phage that display the desired sequences. The digestion process itself can be followed with single-molecule resolution, offering an intriguing opportunity to measure single-molecule enzyme kinetics. The digestion process is also a route to production of non-infective phage particles in which infectivity can be restored by SIP-technology. The aforementioned applications, however, extend beyond the scope of this report and will be described in subsequent reports.

Increased efficiency of ID-display when compared to NT-display can be understood by reviewing the mechanisms by which pIII protein is incorporated into the phage. The role of the pIII protein is termination of phage assembly (Rakonjac and Model, 1998; Rakonjac et al., 1999); the first step in this process is inner-membrane docking and translocation of pIII via the Sec pathway (Rapoza and Webster, 1993). At least two factors can lead to defective processing of pIII: (i) presence of negatively-charged residues or specific residues in specific positions (e.g., Pro in position 2) can inhibit leader peptidase processing (reviewed in Wilson and Finlay, 1998); (ii) the early formation of the loop structure in the membrane in the Sec-pathway follows “positive-inside rule” (von Heijne, 1989) and it is sensitive to the presence of positivelly charged residues in the first 20 amino acids downstream of the signal peptide (von Heijne, 1994). It is known that the transition from low to normal Arg+Lys content occurs in loops made of >60 amino acids (von Heijne, 1994). ID-PhD places the insert over 70 amino acid residues away. Display of Arg-rich sequences is effective in ID-format because the “positive-inside rule” does not apply when charged inserts are remote from the signal sequence. Similarly, display of FLAG sequence is effective because placing the insert in between the domains alleviates problems associated with peptidase processing.

In this study we confirmed ID-insertion of 15 peptide sequences and characterized the amplification for six of these sequences and recognition properties for two sequences. Investigation of a broader class of displayed peptides will be necessary to conclusively describe the behavior of this platform. To this end, cloning and deep-sequencing of several types of peptide libraries in ID-PhD format will be a fruitful direction. We validated the performance of the ID-PhD platform in selection using only a model “library” composed of two clones. Further studies with selection of ligands from a diverse library of peptides in ID-PhD format should be conducted to validate the performance of this platform. The focus of this manuscript is censorship within short peptide libraries and only briefly on protein mini-domains. Censorship in display of larger proteins involves other factors not present in short motifs (e.g., folding). Study of protein display in this system would require a separate investigation.

### Conflict of interest statement

The authors declare that the research was conducted in the absence of any commercial or financial relationships that could be construed as a potential conflict of interest.
