# Supplementary material for: Intra-domain phage display (ID-PhD) of peptides and protein mini-domains censored from canonical pIII phage display
Source: Front Microbiol. 2015 Apr 28;6:340. doi: 10.3389/fmicb.2015.00340 (PMC4412080; doi:10.3389/fmicb.2015.00340)
Supplement: Supplementary file 1 [file DataSheet1.PDF]

Intra-domain phage display (ID-PhD) of peptides and mini-domain proteins censored from canonical pIII phage display.

Katrina F. Tjhung, Frédérique Deiss, Jessica Tran, J. Ying Chou, Ratmir Derda*

Department of Chemistry and Alberta Glycomics Centre, University of Alberta, Edmonton, AB T6G 2G2, Canada

*Corresponding author: ratmir@ualberta.ca

Construction of ID1 and ID2 cloning vectors

Site-directed mutagenesis was used to insert SacI and SpeI restriction enzyme sites into M13KE phage cloning vector to serve as an insertion point for TEVrs-FLAG/mZ synthetic DNA fragments. We used the QuikChange II Site-Directed Mutagenesis kit (Agilent Technologies, Santa Clara, CA, USA).

Mutagenesis primer sequences:

```
Sense (anneal to 3'→5' strand of template):
5'-ctgagggtggtggcgagctcctgacaactagttctgagggtggcg-3'

Antisense (anneal to 5'→3' strand of template):
3'-gactcccaccaccgctcgaggactgttgatcaagactcccaccgc-5'

SacI   SpeI
Blue = complementary to M13KE vector
```

Validation of ID1 and ID2 cloning vectors by sequencing

Sequencing results are given in the reverse complement.

Reverse complement of SacI/SpeI insert: actagttgtcaggagctc

```
CLUSTAL 2.1 multiple sequence alignment

ID1-PhD         ------------------------------------------------------------
ID2-PhD         ------------------------------------------------------------
M13KE           TTAAGACTCCTTATTACGCAGTATGTTAGCAAACGTAGAAAATACATACATAAAGGTGGC 60

ID1-PhD         ------------------------------------------------------------
ID2-PhD         ------------------------------------------------------------
M13KE           AACATATAAAAGAAACGCAAAGACACCACGGAATAAGTTTATTTTGTCACAATCAATAGA 120

ID1-PhD         ------------------------------------------------------------
ID2-PhD         ------------------------------------------------------------
M13KE           AAATTCATATGGTTTACCAGCGCCAAAGACAAAAGGGCGACATTCAACCGATTGAGGGAG 180

ID1-PhD         ------------------------------------------------------------
ID2-PhD         ------------------------------------------------------------
```

```
M13KE           GGAAGGTAAATATTGACGGAAATTATTCATTAAAGGTGAATTATCACCGTCACCGACTTG 240

ID1-PhD         ------------------------------------------------------------
ID2-PhD         ------------------------------------------------------------
M13KE           AGCCATTTGGGAATTAGAGCCAGCAAAATCACCAGTAGCACCATTACCATTAGCAAGGCC 300

ID1-PhD         ------------------------------------------------------------
ID2-PhD         ------------------------------------------------------------
M13KE           GGAAACGTCACCAATGAAACCATCGATAGCAGCACCGTAATCAGTAGCGACAGAATCAAG 360

ID1-PhD         --------------CNGANTGTAGCGCGTTTTCNTCGGCATTTTCGGTCATAGCCCCCTT 46
ID2-PhD         -------------------TGTAGCGCGTTTTCNTCGGCATTTTCGGTCATAGCCCCCTT 41
M13KE           TTTGCCTTTAGCGTCAGACTGTAGCGCGTTTTCATCGGCATTTTCGGTCATAGCCCCCTT 420
                                   ************** **************************

ID1-PhD         ATTAGCGTTTGCCATCTTTTCNTAATCAAAATCACCGGAACCAGAGCCACCACCGGAACC 106
ID2-PhD         ATTAGCGTTTGCCATCTTTTCNTAATCAAAATCACCGGAACCAGAGCCACCACCGGAACC 101
M13KE           ATTAGCGTTTGCCATCTTTTCATAATCAAAATCACCGGAACCAGAGCCACCACCGGAACC 480
                ********************* **************************************

ID1-PhD         GCCTCCCTCAGAGCCGCCACCCTCAGAAC---------------CGCCACCCTCAGAGCC 151
ID2-PhD         GCCTCCCTCAGAGCCGCCACCCTCAGAACTAGTTGTCAGGAGCTCGCCACCCTCAGAGCC 161
M13KE           GCCTCCCTCAGAGCCGCCACCCTCAGAAC---------------CGCCACCCTCAGAGCC 525
                *****************************               ****************

ID1-PhD         ACCACCCTCAGAGCCGCCACCAGAACCACCACCAGAGCCGCCGCCAGCATTGACAGGAGG 211
ID2-PhD         ACCACCCTCAGAGCCGCCACCAGAACCACCACCAGAGCCGCCGCCAGCATTGACAGGAGG 221
M13KE           ACCACCCTCAGAGCCGCCACCAGAACCACCACCAGAGCCGCCGCCAGCATTGACAGGAGG 585
                ************************************************************

ID1-PhD         TTGAGGCAGGTCAGACGATTGGCCTTGATATTCACAAACAAATAAATCCTCATTAAAGCC 271
ID2-PhD         TTGAGGCAGGTCAGACGATTGGCCTTGATATTCACAAACAAATAAATCCTCATTAAAGCC 281
M13KE           TTGAGGCAGGTCAGACGATTGGCCTTGATATTCACAAACAAATAAATCCTCATTAAAGCC 645
                ************************************************************

ID1-PhD         AGAATGGAAAGCGCAGTCTCTGAATTTACCGTTCCAGTAAGCGTCATACATGGCTTTTGA 331
ID2-PhD         AGAATGGAAAGCGCAGTCTCTGAATTTACCGTTCCAGTAAGCGTCATACATGGCTTTTGA 341
M13KE           AGAATGGAAAGCGCAGTCTCTGAATTTACCGTTCCAGTAAGCGTCATACATGGCTTTTGA 705
                ************************************************************

ID1-PhD         TGATACAGGAGTGTACTGGTAATAAGTTTTAACGGGGTCAGTGCCTTGAGTAACAGTGCC 391
ID2-PhD         TGATACAGGAGTGTACTGGTAATAAGTTTTAACGGGGTCAGTGCCTTGAGTAACAGTGCC 401
M13KE           TGATACAGGAGTGTACTGGTAATAAGTTTTAACGGGGTCAGTGCCTTGAGTAACAGTGCC 765
                ************************************************************

ID1-PhD         CGTATAAACAGTTAATGCCCCCTGCCTATTTCGGAACCTATTATTCTGAAACATGAAAGT 451
ID2-PhD         CGTATAAACAGTTAATGCCCCCTGCCTATTTCGGAACCTATTATTCTGAAACATGAAAGT 461
M13KE           CGTATAAACAGTTAATGCCCCCTGCCTATTTCGGAACCTATTATTCTGAAACATGAAAGT 825
                ************************************************************

ID1-PhD         ATTAAGAGGCTGAGACTCCTCAAGAGAAGGATTAGGATTAGCGGGGTTTTGCTCAGTACC 511
ID2-PhD         ATTAAGAGGCTGAGACTCCTCAAGAGAAGGATTAGGATTAGCGGGGTTTTGCTCAGTACC 521
M13KE           ATTAAGAGGCTGAGACTCCTCAAGAGAAGGATTAGGATTAGCGGGGTTTTGCTCAGTACC 885
                ************************************************************

ID1-PhD         AGGCGGATAAGTGCCGTCGAGAGGGTTGATATAAGTATAGCCCGGAATAGGTGTATCACC 571
ID2-PhD         AGGCGGATAAGTGCCGTCGAGAGGGTTGATATAAGTATAGCCCGGAATAGGTGTATCACC 581
M13KE           AGGCGGATAAGTGCCGTCGAGAGGGTTGATATAAGTATAGCCCGGAATAGGTGTATCACC 945
                ************************************************************

ID1-PhD         GTACTCAGGAGGTTTAGTACCGCCACCCTCAGAACCGCCACCCTCAGAACTAGTTGTCAG 631
ID2-PhD         GTACTCAGGAGGTTTAGTACCGCCACCCTCAGAACCGCCACCCTCAGAACCGCCACCCTC 641
M13KE           GTACTCAGGAGGTTTAGTACCGCCACCCTCAGAACCGCCACCCTCAGAACCGCCACCCTC 1005
                *************************************************  .  :  *:

ID1-PhD         GAGCTCGCCACCACCCTCATTTTCAGGGATAGCAAGCCCAATAGGAACCCATGTACCGTA 691
ID2-PhD         AG---AGCCACCACCCTCATTTTCAGGGATAGCAAGCCCAATAGGAACCCATGTACCGTA 698
M13KE           AG---AGCCACCACCCTCATTTTCAGGGATAGCAAGCCCAATAGGAACCCATGTACCGTA 1062
                ..   .******************************************************

ID1-PhD         ACACTGAGTTTCGTCACCAGTACAAACTACAACGCCTGTAGCATTCCACAGACAGCCCTC 751
ID2-PhD         ACACTGAGTTTCGTCACCAGTACAAACTACAACGCCTGTAGCATTCCACAGACAGCCCTC 758
M13KE           ACACTGAGTTTCGTCACCAGTACAAACTACAACGCCTGTAGCATTCCACAGACAGCCCTC 1122
                ************************************************************

ID1-PhD         ATAGTTAGCGTAACGATCTAAAGTTTTGTCGTCTTTCCAGACGTTAGTAAATGAATTTTC 811
ID2-PhD         ATAGTTAGCGTAACGATCTAAAGTTTTGTCGTCTTTCCAGACGTTAGTAAATGAATTTTC 818
M13KE           ATAGTTAGCGTAACGATCTAAAGTTTTGTCGTCTTTCCAGACGTTAGTAAATGAATTTTC 1182
                ************************************************************

ID1-PhD         TGTATGGGATTTTGCTAAACAACTTTCAANAGTTTCNNCCGAACCTCCACCCGNNTGANA 871
ID2-PhD         TGTATGGGATTTTGCTAAACAACTTTCAACAGTTTCGGCCGAACCTCCACCNNCATGATA 878
M13KE           TGTATGGGATTTTGCTAAACAACTTTCAACAGTTTCGGCCG------------------- 1223
                ***************************** ******..***
```

Inserted constructs (ID-cloning)
To test our ID-PhD system, we inserted the following DNA constructs.
ID2-vector was used for ID, RAE and X4 constructs and M13KE vector was used for the NT-FLAG-TEVrs construct. Restriction sites: SacI SpeI KpnI EagI

ID-TEVrs-FLAG
```
5'-GGCCCGgagctcGAAAACCTGTATTTTCAGTCGGACTACAAGGACGACGATGACAAGactagtGCGCCG-3'
            E  N  L  Y  F  Q  S  D  Y  K  D  D  D  D  K
```
ID-mZ-TEVrs
```
5'-GGCCCGgagctctttaacatgcagcagcagcgccgcttttatgaagcgctgcatgatccgaacctgaacgaagaac
            F  N  M  Q  Q  Q  R  R  F  Y  E  A  L  H  D  P  N  L  N  E  E
agcgcaacgcgaaaattaaaagcattcgcgatgatGAAAACCTGTATTTTCAGTCGactagtGCGCCG -3'
Q  R  N  A  K  I  K  S  I  R  D  D  E  N  L  Y  F  Q  S
```

RAE1
```
5'-GGCCCGgagctcGAAAACCTGTATTTTCAGTCGGCGCGTGAGGCACGTCGCGCAGAGAGGGAAactagtGCGCCGATCGC-3'
            E  N  L  Y  F  Q  S  A  R  E  A  R  R  A  E  R  E
```
RAE2
```
5'-GGCCCGgagctcGAAAACCTGTATTTTCAGTCGAGGAGGGAAGCGGCTGCCGAGAGAGCGCGGactagtGCGCCGATCGC-3'
            E  N  L  Y  F  Q  S  R  R  E  A  A  A  E  R  A  R
```
RAE3
```
5'-GGCCCGgagctcGAAAACCTGTATTTTCAGTCGGCTGCGGCGAGAAGAAGAGCCGAGCGCGCAactagtGCGCCGATCGC-3'
            E  N  L  Y  F  Q  S  A  A  A  R  R  R  A  E  R  A
```
RAE4
```
5'-GGCCCGgagctcGAAAACCTGTATTTTCAGTCGCGTGCCGCTGCTAGAAGAGCCAGGGCTGCCactagtGCGCCGATCGC-3'
            E  N  L  Y  F  Q  S  R  A  A  A  R  R  A  R  A  A
```
X4
```
5'-GGCCCGgagctcGAAAACCTGTATTTTCAGTCGNNKNNKNNKNNKactagtGCGCCGATCGC-3'
            E  N  L  Y  F  Q  S  X  X  X  X
```

Extension primer to extend ssDNA to dsDNA:

5'-CGGCGCACTAGTCTTGTCATC-3' (for TD-TEV-FLAG)
5'-GCGATCGGCGCACTAGT –3' (for RAE and X4 constructs)

Sequencing primer for ID-constructs: 5' – CACCGTAATCAGTAGCGACA – 3'

Inserted constructs (NT-cloning)

NT-FLAG-TEVrs
```
5'-CATGCCCGGGTACCTTTCTATTCTCACTCTTCTGACTACAAGGACGACGATGACAAGGAAAACCTGTATTTTCAGTCG
                                  D  Y  K  D  D  D  D  K  E  N  L  Y  F  Q  S
GGTGGAGGTTCGGCCGGGCGC -3'
```

Extension primer and "96-sequencing primer" were from Ph.D.™-12 Phage Display Peptide Library Kit (New England Biolabs)

Alignment of amino acid sequences of ID1/ID2-FLAG-TEVrs insert

Amino acid sequence of TEVrs-FLAG insert: ELENLYFQSDYKDDDDKTS

```
ID2-FLAG        VKKLLFAIPLVVPFYSHSAETVESCLAKSHTENSFTNVWKDDKTLDRYANYEGCLWNATG 60
ID1-FLAG        VKKLLFAIPLVVPFYSHSAETVESCLAKSHTENSFTNVWKDDKTLDRYANYEGCLWNATG 60
                ************************************************************

ID2-FLAG        VVVCTGDETQCYGTWVPIGLAIPENEGGGELENLYFQSDYKDDDDKTSSEGGGSEGGGTK 120
ID1-FLAG        VVVCTGDETQCYGTWVPIGLAIPENEGGG--------------SEGGGSEGGGSEGGGTK 106
                *****************************              .:  .************

ID2-FLAG        PPEYGDTPIPGYTYINPLDGTYPPGTEQNPANPNPSLEESQPLNTFMETFQNNRFRNRQG 180
ID1-FLAG        PPEYGDTPIPGYTYINPLDGTYPPGTEQNPANPNPSLEESQPLNTFMETFQNNRFRNRQG 166
                ************************************************************

ID2-FLAG        ALTVYTGTVTQGTDPVKTYYQYTPVSSKAMETYDAYWNGKFRDCAFHSGFNEDLFVCEYQ 240
ID1-FLAG        ALTVYTGTVTQGTDPVKTYYQYTPVSSKAMETYDAYWNGKFRDCAFHSGFNEDLFVCEYQ 226
                ************************************************************

ID2-FLAG        GQSSDLPQPPVNAGGGSGGGSGGGSEGGG-------------------SEGGGSEGGGSE 281
ID1-FLAG        GQSSDLPQPPVNAGGGSGGGSGGGSEGGGELENLYFQSDYKDDDDKTSSEGGGSEGGGSE 286
                *****************************                   ************

ID2-FLAG        GGGSGGGSGSGDFDYEKMETANANKGAMETTENADENALQSDAKGKLDSVATDYGAAIDG 341
ID1-FLAG        GGGSGGGSGSGDFDYEKMETANANKGAMETTENADENALQSDAKGKLDSVATDYGAAIDG 346
                ************************************************************

ID2-FLAG        FIGDVSGLANGNGATGDFAGSNSQMETAQVGDGDNSPLMETNNFRQYLPSLPQSVECRPF 401
ID1-FLAG        FIGDVSGLANGNGATGDFAGSNSQMETAQVGDGDNSPLMETNNFRQYLPSLPQSVECRPF 406
                ************************************************************

ID2-FLAG        VFGAGKPYEFSIDCDKINLFRGVFAFLLYVATFMETYVFSTFANILRNKESStop 456
ID1-FLAG        VFGAGKPYEFSIDCDKINLFRGVFAFLLYVATFMETYVFSTFANILRNKESStop 461
                ********************************************************
```

Validation of mZ-TEV insert by sequencing

Alignment of expected mZ-TEV sequence with sequenced ID2-mZ vector, showing insertion between SacI and SpeI restriction sites.

```
mZ-TEV          ---------------TTTAACATGCAGCAGCAGCGCCGCTTTTATGAAGCGCTGCATGAT 45
ID2-mZ          GAGGGTGGCGAGCTCTTTAACATGCAGCAGCAGCGCCGCTTTTATGAAGCGCTGCATGAT 1067
                               *********************************************

mZ-TEV          CCGAACCTGAACGAAGAACAGCGCAACGCGAAAATTAAAAGCATTCGCGATGATGAAAAC 105
ID2-mZ          CCGAACCTGAACGAAGAACAGCGCAACGCGAAAATTAAAAGCATTCGCGATGATGAAAAC 1127
                ************************************************************

mZ-TEV          CTGTATTTTCAGTCG--------------------------------------------- 120
ID2-mZ          CTGTATTTTCAGTCGACTAGTTCTGAGGGTGGCGGCTCTGAGGGAGGCGGTTCCGGTGGT 1187
                ***************
```

Validation of “RAE” and library insert by sequencing

#	name	Sequence<br>N1, N2 and CT domains, linkers and inserted sequences are color-coded.
1	AREARRAERE	WKDDKTLDRYANYEGCLWNATGVVVCTGDETQCYGTWVPIGLAIPENEGGGELENLYFQSAREAR<br>RAERETSSEGGGSEGGGTKPPEYGDTPIPGYTYINPLDGTYPPGTEQNPANPNPSLEESQPLNTF<br>MFQNNRFRNRQGALTVYTGTVTQGTDPVKTYYQYTPVSSKAMYDAYWNGKFRDCAFHSGFNEDLF<br>VCEYQGQSSDLPQPPVNAGGGSGGGSGGGSEGGGSEGGGSEGGGSEGGGSGGGSGSGDFDYEKMA<br>NANKGAMTENA
2	AREARRAERE	WKDDKTLDRYANYEGCLWNATGVVVCTGDETQCYGTWVPIGLAIPENEGGGELENLYFQSRREAA<br>AERARTSSEGGGSEGGGTKPPEYGDTPIPGYTYINPLDGTYPPGTEQNPANPNPSLEESQPLNTF<br>MFQNNRFRNRQGALTVYTGTVTQGTDPVKTYYQYTPVSSKAMYDAYWNGKFRDCAFHSGFNEDLF<br>VCEYQGQSSDLPQPPVNAGGGSGGGSGGGSEGGGSEGGGSEGGGSEGGGSGGGSGSGDFDYEKMA<br>NANKGAMTENA
3	AAARRRAERA	ENSFTNVWKDDKTLDRYANYEGCLWNATGVVVCTGDETQCYGTWVPIGLAIPENEGGGELENLYF<br>QSAAARRRAERATSSEGGGSEGGGTKPPEYGDTPIPGYTYINPLDGTYPPGTEQNPANPNPSLEE<br>SQPLNTFMFQNNRFRNRQGALTVYTGTVTQGTDPVKTYYQYTPVSSKAMYDAYWNGKFRDCAFHS<br>GFNEDLFVCEYQGQSSDLPQPPVNAGGGSGGGSGGGSEGGGSEGGGSEGGGSEGGGSGGGSGSGD<br>FDYEKMANANKGAMTENA
4	RAAARGARAA	ENSFTNVWKDDKTLDRYANYEGCLWNATGVVVCTGDETQCYGTWVPIGLAIPENEGGGELENLYF<br>QSRAAARGARAATSSEGGGSEGGGTKPPEYGDTPIPGYTYINPLDGTYPPGTEQNPANPNPSLEE<br>SQPLNTFMFQNNRFRNRQGALTVYTGTVTQGTDPVKTYYQYTPVSSKAMYDAYWNGKFRDCAFHS<br>GFNEDLFVCEYQGQSSDLPQPPVNAGGGSGGGSGGGSEGGGSEGGGSEGGGSEGGGSGGGSGSGD<br>FDYEKMANANKGAMTENA
5	Example of library clone	GVVVCTGDETQCYGTWVPIGLAIPENEGGGELENLYFQSFGRNTSSEGGGSEGGGTKPPEYGDTP<br>IPGYTYINPLDGTYPPGTEQNPANPNPSLEESQPLNTFMFQNNRFRNRQGALTVYTGTVTQGTDP<br>VKTYYQYTPVSSKAMYDAYWNGKFRDCAFHSGFNEDLFVCEYQGQSSDLPQPPVNAGGGSGGGSG<br>GGSEGGGSEGGGSEGGGSEGGGSGGGSGSGDFDYEKMANANKGAMTE

Validation of FLAG insert by sequencing

#	name	Sequence<br>N1 domain, linkers, leader and inserted sequences are color-coded.
1	NT-FLAG-TEVrs	VKKLLFAIPLVVPFYSHSSDYKDDDDKENLYFQSGGGSAETVESCLAKSHTENS
